# ﻿Two new species of cave-adapted pseudoscorpions (Pseudoscorpiones, Chthoniidae) from Yunnan, China

**DOI:** 10.3897/zookeys.1097.82527

**Published:** 2022-04-20

**Authors:** Yanmeng Hou, Zhizhong Gao, Feng Zhang

**Affiliations:** 1 The Key Laboratory of Zoological Systematics and Application, Institute of Life Science and Green Development, Hebei University, Baoding, Hebei 071002, China Hebei University Baoding China; 2 Department of Biology, Xinzhou Teachers University, Xinzhou 034000, Shanxi Province, China Xinzhou Teachers University Xinzhou China

**Keywords:** Karst biotope, *Lagynochthonius*, taxonomy, troglobionts, *Tyrannochthonius*

## Abstract

Two new cave-adapted pseudoscorpion species belonging to the family Chthoniidae are described: *Tyrannochthoniuspandus***sp. nov.** from Biyu Cave (Yunnan: Luxi) and *Lagynochthoniuslaoxueyanensis***sp. nov.** from Laoxueyan Cave (Yunnan: Yanshan). Both of them, collected from the dark zone of caves, are highly troglomorphic species.

## ﻿Introduction

China has the largest karst biotopes in the world, with the karst area reaching 3.44 million km^2^, accounting for about one-third of the country’s land area, and contains tens of thousands of karst caves, which are rich in animal resources (Zhao et al. 2015). Yunnan, located in southwest China, is one of the provinces with the most widely distributed karst landforms (11.09 × 10^4^ km^2^), especially in the eastern portion of Yunnan (Wang 2001). So far, at least 742 cave-dwelling species have been identified in China, and nearly 15% of them are from Yunnan (Latella 2019). Subterranean-adapted pseudoscorpions are one of the representative groups of cave-dwelling arthropods. They are usually eyeless, have elongate appendages and can be easily found on cave walls or under rocks. To date, 33 cave-dwelling pseudoscorpion species, representing three families (Chthoniidae, Neobisiidae, Chernetidae), have been described from China. Among them, eight species are known from Yunnan (Schawaller 1995; Mahnert 2003, 2009; Mahnert and Li 2016; Gao et al. 2017; Li et al. 2017; Gao et al. 2018; Li et al. 2019; Feng et al. 2020; Gao et al. 2020; Zhang et al. 2020).

The genus *Tyrannochthonius* Chamberlin, 1929 contains 145 species, with at least 52 species occurring in caves, and is distributed in all continents except Antarctica (WPC 2022). This genus can be diagnosed as follows (see Material and methods for explanation of abbreviations): trichobothrium *sb* situated midway between *st* and *b*, or closer to *st*; trichobothria *ib* and *isb* situated close together in a median or sub-basal position on the dorsum of the chelal hand; chelal hand not distally constricted and the movable finger without a complex or strongly sclerotized apodeme at the base; fixed finger usually with one large, medial acuminate spine-like seta at its base, but can be reduced or absent in some cave-dwelling species; coxal spines generally long and present on coxae II only; epistome pointed, triangular or rounded, inconspicuous and usually with 2 closely-flanking setae at its base (Chamberlin 1962; Muchmore 1984, 1991; Muchmore and Chamberlin 1995; Edward and Harvey 2008). So far, nine species and one subspecies of this genus have been described from China, of which six are exclusively known from karst caves: *T.akaelus* Mahnert, 2009 from Sichuan, *T.ganshuanensis* Mahnert, 2009 from Sichuan and Hubei, *T.antridraconis* Mahnert, 2009 from Sichuan, *T.chixingi* Gao, Wynne & Zhang, 2018 from Guangxi, *T.harveyi* Gao, Zhang & Chen, 2020 and *T.zhai* Gao, Zhang & Chen, 2020 from Guizhou. All species are troglobites without eyes (Mahnert 2009; Gao et al. 2018; Gao et al. 2020; WPC 2022).

The genus *Lagynochthonius* Beier, 1951 was erected by Beier (1951) as a subgenus of *Tyrannochthonius*, but was later elevated to generic status by Chamberlin (1962). The genus is diagnosed by trichobothrium *sb* situated midway between *st* and *b*, or closer to *st*; trichobothria *ib* and *isb* situated close together in a median or sub-basal position on the dorsum of the chelal hand; coxal spines generally long and present on coxae II only; chelal hand distally constricted (or flask-shaped) and movable finger with complex or strongly sclerotized apodeme at its base and the modified tooth (*td*) of the fixed chelal finger displaced onto the dorso-antiaxial face (Chamberlin 1962; Harvey 1989; Muchmore 1991; Judson 2007; Edward and Harvey 2008). At present, this genus contains 55 species (seven species living in caves) distributed in Asia, Australia, Africa and America. Eight species of this genus have been described from China, of which only one is exclusively known from karst caves: *L.bailongtanensis* Li, Liu & Shi, 2019 from Yunnan (Li et al. 2019; WPC 2022).

Two new troglomorphic species of Chthoniidae have been recently found from the karst caves survey in Yunnan in 2021. These species are here described.

## ﻿Materials and methods

The specimens examined for this study are preserved in 75% alcohol and deposited in the Museum of Hebei University (**MHBU**) (Baoding, China) and the Museum of Southwest University (**MSWU**) (Chongqing, China). Photographs, drawings and measurements were taken using a Leica M205A stereo-microscope equipped with a Leica DFC550 Camera and the Inkscape software (Ver. 1.0.2.0). Detailed examination was carried out with an Olympus BX53 general optical microscope. Images were edited and formatted using Adobe Photoshop 2022.

Terminology and measurements follow Chamberlin (1931) with some minor modifications to the terminology of trichobothria (Harvey 1992; Judson 2007) and chelicera (Judson 2007). The chela and chelal hand are measured in lateral view and others taken in dorsal view. All measurements are given in mm unless noted otherwise. Proportions and measurements of pedipalps and carapace correspond to length/width, those of legs to length/depth.

The following abbreviations are used in the text:

**b** basal trichobothrium;

**sb** sub-basal trichobothrium;

**st** sub-terminal trichobothrium;

**t** terminal trichobothrium trichobothrium;

**ib** interior basal trichobothrium;

**isb** interior sub-basal trichobothrium;

**ist** interior sub-terminal trichobothrium;

**it** interior terminal trichobothrium;

**eb** exterior basal trichobothrium;

**esb** exterior sub-basal trichobothrium;

**est** exterior sub-terminal trichobothrium;

**et** exterior terminal trichobothrium;

**dx** duplex trichobothria;

**td** modified tooth.

## ﻿Taxonomy

### ﻿Chthoniidae Daday, 1889

#### ﻿ Tyrannochthonius

Taxon classificationAnimaliaPseudoscorpionesChthoniidae

Chamberlin, 1929

13E03AE6-EE91-5089-B1CB-3636D541C2F2

##### Type species.

*Chthoniusterribilis* With, 1906, by original designation.

##### Diagnosis.

See Edward and Harvey (2008).

#### ﻿ Tyrannochthonius pandus sp. nov.

Taxon classificationAnimaliaPseudoscorpionesChthoniidae

385C611D-235E-5B0D-9B1E-4E54164EA1B9

http://zoobank.org/D9B22241-9699-41ED-936C-5169456BD61A

Chinese name. 弯指暴伪蝎 Figs 2 , 3 , 4 , 5

##### Type material.

**Figure 1.**
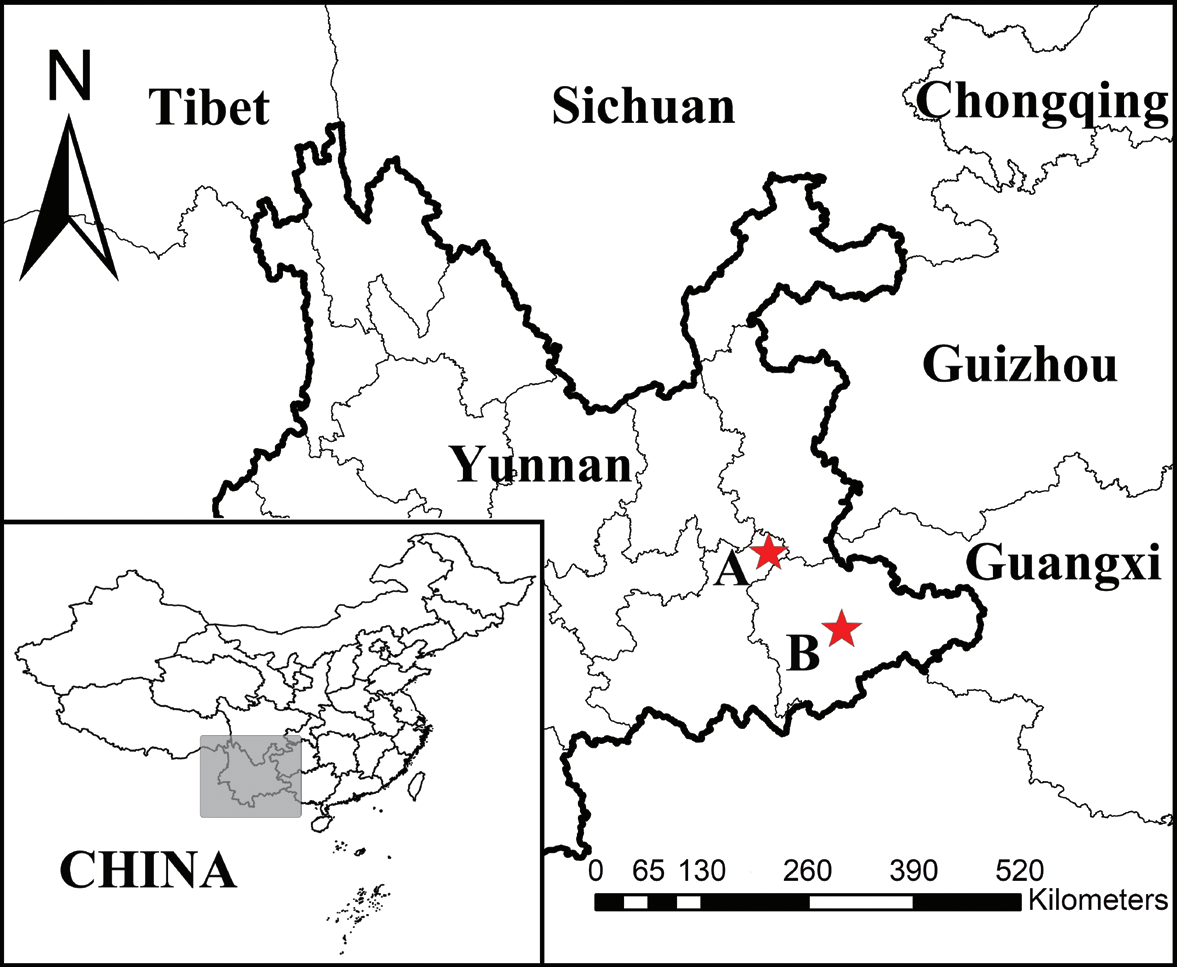
Study area, general cave locations, and type locality for each species, Yunnan Province, China **A** Biyu Cave, *Tyrannochthoniuspandus* sp. nov. **B** Laoxueyan Cave, *Lagynochthoniuslaoxueyanensis* sp. nov.

(Figs 1A, 6) ***Holotype***: China • ♂; Yunnan Province, Luxi County, Luyuandong Village, the Ancient Alu Cave National Park of China, Biyu Cave; 24°34.01'N, 103°45.16'E; 1722 m a.s.l.; 13 Oct. 2021; Zegang Feng, Yanmeng Hou, Lu Zhang and Liu Fu leg.; dark zone; Ps.-**MHBU**-HBUARA#2021-438-01. ***Paratype***: • 1♀; the same data as the holotype; Ps.-**MSWU**-HBUARA#2021-438-02.

##### Diagnosis.

Moderately sized troglomorphic species with elongate appendages; carapace without eyes or eyespots; anterior margin of carapace gently serrate, epistome small, pointed, triangular, with 2 setae flanking base; posterior margin of carapace with 2 setae; tergites I–III with 2 setae; lacking chemosensory setae on dorsum of chelal hand; chelal fingers distinctly curved in dorsal view, with numerous large, gently curved, well-spaced teeth.

##### Etymology.

The specific name is derived from the Latin word “*pandus*”, meaning curved, refers to the curved chelal finger.

##### Description.

**Figure 2.**
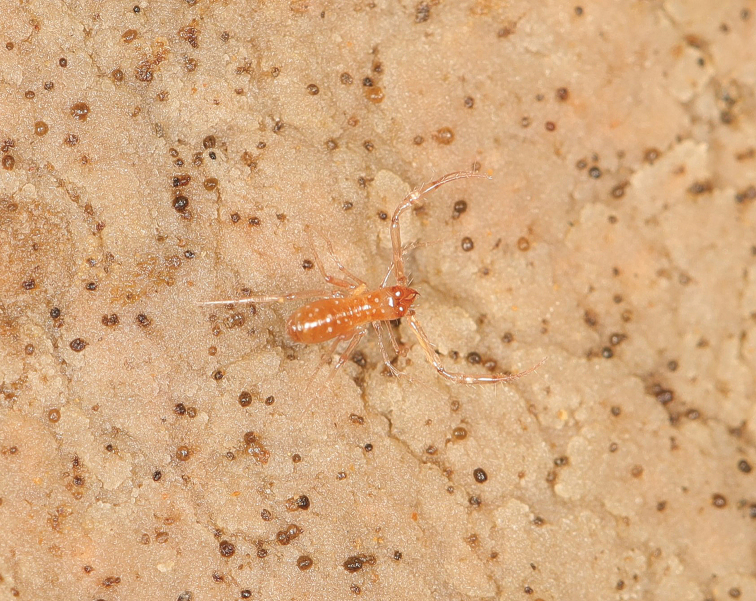
*Tyrannochthoniuspandus* sp. nov., male habitus. Photographed when it crawled on stony natural habitat.

**Figure 3.**
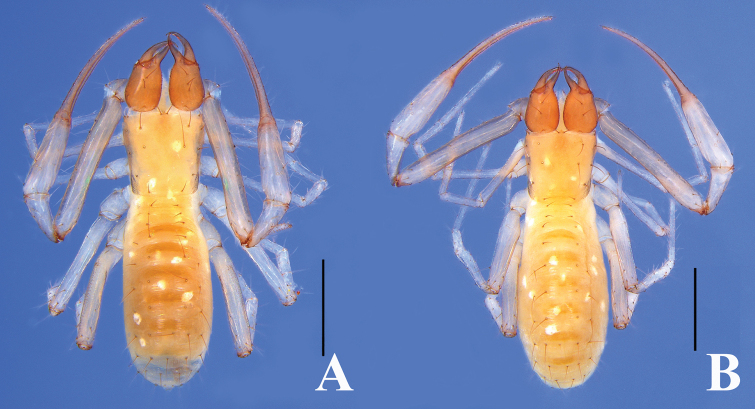
*Tyrannochthoniuspandus* sp. nov. **A** holotype male, dorsal view **B** paratype female, dorsal view. Scale bars: 0.5 mm.

**Figure 4.**
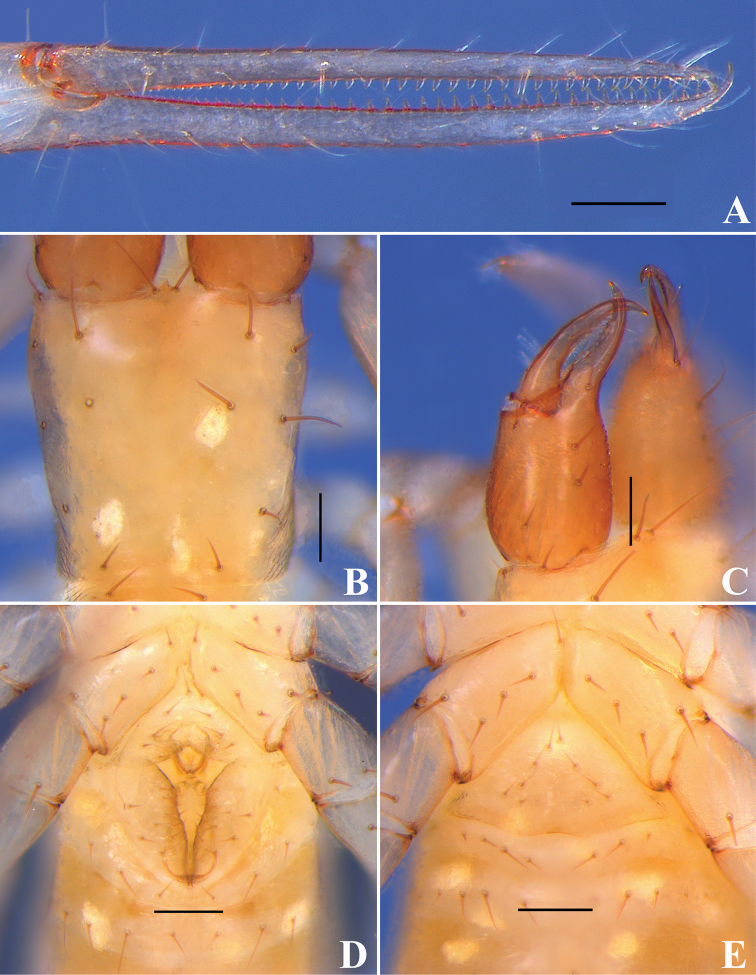
*Tyrannochthoniuspandus* sp. nov., holotype male (**A–D**), paratype female (**E**) **A** chelal fingers (lateral view) **B** carapace (dorsal view) **C** left chelicera (dorsal view) **D** male genital area (ventral view) **E** female genital area (ventral view). Scale bars: 0.1 mm.

**Figure 5.**
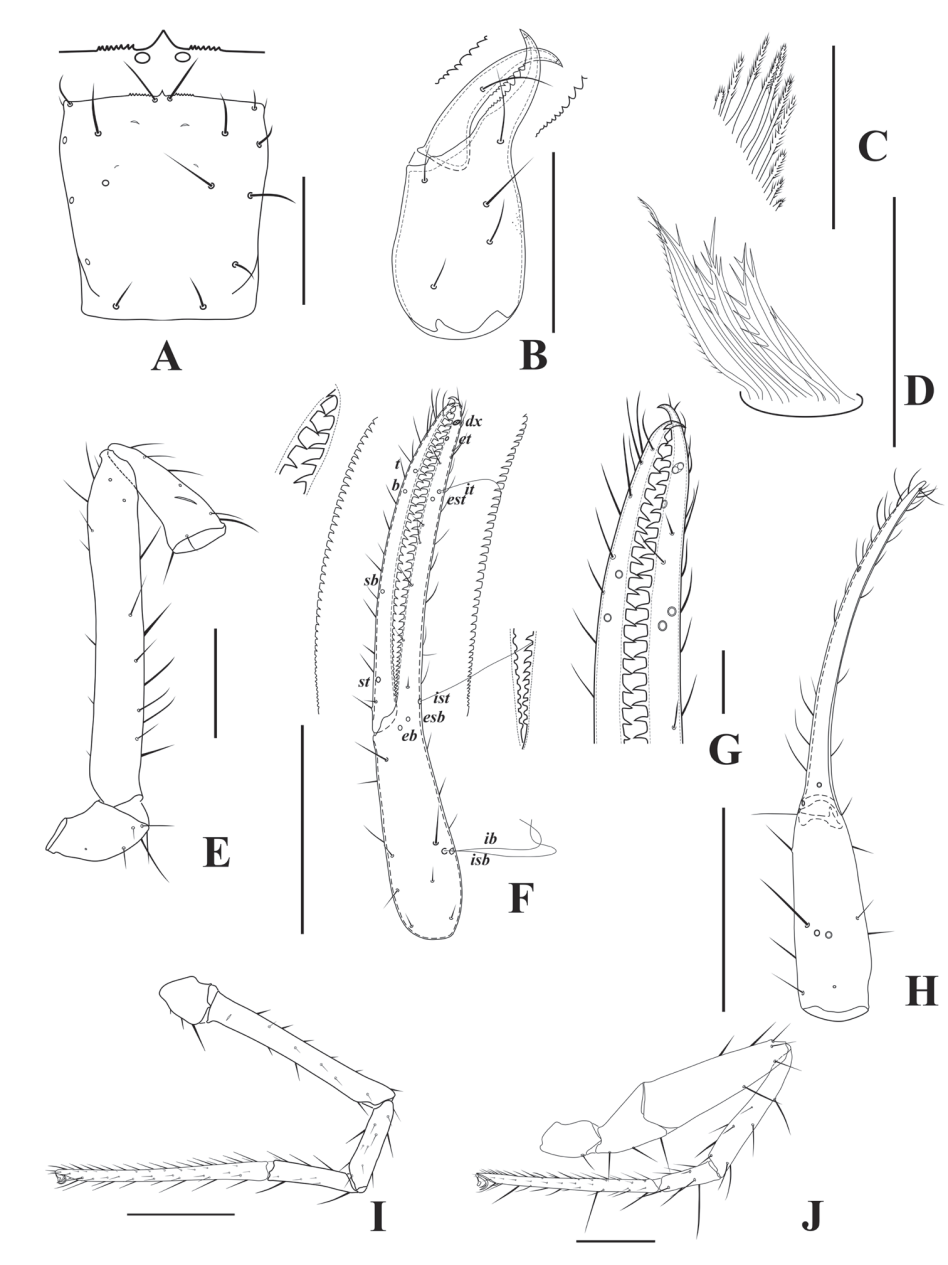
*Tyrannochthoniuspandus* sp. nov., holotype male **A** carapace (dorsal view) with a detail of anterior margin **B** left chelicera (dorsal view) with details of teeth **C** coxal spines on coxae II (ventral view) **D** rallum **E** left pedipalp (minus chela, dorsal view) **F** left chela (lateral view) with details of teeth and with trichobothrial pattern (abbreviations explained in Material and methods) **G** finger tips of chela (lateral view) **H** left chela (dorsal view) **I** leg I (lateral view) **J** leg IV (lateral view). Scale bars: 0.25 mm (**A–B, E–F, H–J**); 0.10 mm (**C–D, G**).

**Adult male** (Figs 2, 3A, 4A–D, 5). ***Color***: generally pale yellow, chelicerae, pedipalps and tergites slightly darker, soft parts pale (Figs 2, 3A). ***Cephalothorax*** (Figs 4B, 5A): carapace 1.07 times longer than broad, gently narrowed posteriorly, surface smooth; anterior margin slightly serrate; without any traces of eyes; epistome very pointed and small, triangular, with 2 setae flanking base; with 18 setae arranged s4s: 4: 4: 2: 2, most setae heavy, long and gently curved, anterolateral setae much shorter than others; without furrows but with 4 lyrifissures (Fig. 5A). Chaetotaxy of coxae: P 3, I 3, II 4, III 5, IV 5; manducatory process with two acuminate distal setae, anterior seta less than 1/2 length of medial seta; apex of coxa I with small, rounded anteromedial process; coxae II with 10 terminally indented coxal spines on each side, set as an oblique row, longer spines present in the middle of the row, becoming shorter distally and proximally and incised for about half their length (Fig. 5C); intercoxal tubercle absent; without sub-oral seta. ***Chelicera*** (Figs 4C, 5B): large, about as long as carapace, 2.44 times longer than broad; 5 setae on hand, all setae acuminate, ventrobasal seta shorter than others; movable finger with one medial seta. Cheliceral palm with moderate hispid granulation dorsally. Both fingers well provided with teeth, fixed finger with 11 teeth, distal one largest; movable finger with 8 retrorse continuous small teeth; galea completely vestigial (Fig. 5B). Rallum with 8 blades, the distal one longest and recumbent basally, with fine barbules and slightly set apart from the other blades, latter tightly grouped and with long pinnae, some of which are subdivided (Fig. 5D). ***Pedipalp*** (Figs 4A, 5E–H): surface of palpal segments smooth; setae generally long and acuminate; femur 7.36, patella 2.55, chela 7.47, hand 2.80 times longer than deep; movable finger 1.71 times longer than hand and 0.64 times longer than chela, without large basal apodeme, only slightly sclerotized section present. Femur and dorsal hand without tactile setae but with 1 distal lyrifissure on patella (Fig. 5E). Fixed chelal finger and hand with 8 trichobothria, movable chelal finger with 4 trichobothria, *ib* and *isb* situated close together, submedially on dorsum of chelal hand; *eb*, *esb* and *ist* forming a straight oblique row at base of fixed chelal finger; *it* slightly distal to *est*, situated subdistally; *et* slightly near to tip of fixed finger, very close to chelal teeth; *dx* situated distal to *et*; *sb* situated closer to *st* than to *b*; *b* and *t* situated subdistally, *b* situated at same level as *est*; *t* situated distal to *est* (Fig. 5F). Microsetae (chemosensory setae) absent on hand and both chelal fingers. Sensilla absent. A tiny antiaxial lyrifissure present at base of fixed finger (slightly distal to *ist*). Both chelal fingers with a row of teeth, homodentate, spaced regularly along the margin, larger teeth present in the middle of the row, becoming smaller distally and proximally: fixed finger with 45 large, gently curved, well-spaced teeth, without intercalary teeth; movable finger with 44 small (slightly smaller than the teeth on fixed finger), retrorse, gently curved and well-spaced teeth (Fig. 5F). Chelal fingers distinctly curved in dorsal view (Fig. 5H). ***Opisthosoma***: generally typical; pleural membrane finely granulate. Tergites and sternites undivided; setae uniseriate and acuminate. Tergal chaetotaxy I–XII: 2: 2: 2: 3: 4: 4: 4: 5: 5: 4: T2T: 0; tergites VIII and IX each with an unpaired median seta. Sternal chaetotaxy IV–XII: 10: 10: 9: 9: 9: 9: 8: 0: 2; sternites VI–IX with unpaired median seta. Anterior genital operculum with 10 setae, genital opening slit-like, with 15 marginal setae on each side (Fig. 4D). ***Legs*** (Fig. 5I, J): generally typical, long and slender. Femur of leg I 1.92 times longer than patella and with 1 lyrifissure at the base of femur; tarsus 2.50 times longer than tibia. Femoropatella of leg IV 3.67 times longer than deep; tibia 6.29 times longer than deep; with basal tactile setae on both tarsal segments: basitarsus 4.00 times longer than deep (TS = 0.35), telotarsus 14.50 times longer than deep and 2.90 times longer than basitarsus (TS = 0.33). Setae of leg I (trochanter to tibia) 3: 13: 12: 9, setae of leg IV (trochanter to basitarsus) 1: 3: 6: 9: 8. Arolium slightly shorter than the claws, not divided; claws simple. ***Dimensions of male holotype*** (length/width or, in the case of the legs, length/depth in mm; ratios in parentheses). Body length 1.41. Pedipalps: trochanter 0.18/0.13 (1.38), femur 0.81/0.11 (7.36), patella 0.28/0.11 (2.55), chela 1.12/0.15 (7.47), hand length 0.42/0.15 (2.80), movable finger length 0.72. Chelicera 0.44/0.18 (2.44), movable finger length 0.24. Carapace 0.45/0.42 (1.07). Leg I: trochanter 0.13/0.11 (1.18), femur 0.46/0.06 (7.67), patella 0.24/0.05 (4.80), tibia 0.20/0.04 (5.00), tarsus 0.50/0.04 (12.50). Leg IV: trochanter 0.20/0.11 (1.82), femoropatella 0.66/0.18 (3.67), tibia 0.44/0.07 (6.29), basitarsus 0.20/0.05 (4.00), telotarsus 0.58/0.04 (14.50).

**Adult female** (Figs 3B, 4E). Mostly same as males. Anterior genital operculum with 10 setae plus 7 setae on posterior margin. Body length 1.67. Pedipalps: trochanter 0.24/0.13 (1.85), femur 0.88/0.13 (6.77), patella 0.28/0.11 (2.55), chela 1.20/0.17 (7.06), hand length 0.49/0.17 (2.88), movable finger length 0.76. Chelicera 0.45/0.21 (2.14), movable finger length 0.23. Carapace 0.46/0.46 (1.00). Leg I: trochanter 0.14/0.11 (1.27), femur 0.50/0.06 (8.33), patella 0.26/0.06 (4.33), tibia 0.21/0.05 (4.20), tarsus 0.53/0.04 (13.25). Leg IV: trochanter 0.21/0.12 (1.75), femoropatella 0.70/0.19 (3.68), tibia 0.46/0.07 (6.57), basitarsus 0.21/0.06 (3.50), telotarsus 0.62/0.04 (15.50).

##### Remarks.

Compared with the other six cave-dwelling species of the genus in China, *Tyrannochthoniuspandus* sp. nov. is most similar to *T.ganshuanensis* in having only 2 setae on tergites I–III, the same chaetotaxy of carapace and triangular, a small epistome, but differs in the shape of teeth on chelal fingers (large, gently curved, well-spaced teeth, without intercalary teeth in *T.pandus*, but with pointed, well-spaced and intercalary teeth in *T.ganshuanensis*), the relative position of the trichobothria on the movable chelal finger (*sb* situated closer to *st* than to *b* in *T.pandus*, but *sb* situated closer to *b* in *T.ganshuanensis*). *Tyrannochthoniuspandus* sp. nov. can be easily separated from *T.akaleus* by a smaller body size (1.67 vs. 2.10 mm in female), the teeth pattern on chelal fingers (intercalary teeth absent in *T.pandus*, but present in *T.akaleus*); from *T.harveyi* by the different setae number on the anterior and posterior margins of the carapace (*T.pandus* with 6 and 2 setae, respectively, but *T.harveyi* with 4 and 4 setae, respectively), the shape of the epistome (long and pointed in *T.pandus*, but rounded and inconspicuous in *T.harveyi*), the number of rallar blades (8 in *T.pandus*, but 6 in *T.harveyi*); and from *T.zhai*, *T.chixingi* and *T.antridraconis* by the number of setae on the anterior tergites (tergites I–III with 2 setae in *T.pandus*, but the other three with 4 setae). In addition, compared to the new species, *T.zhai* differs by the shorter body length (1.40 vs. 1.67 mm in female) and lacking an epistome; *T.chixingi* and *T.antridraconis* differs from the new species also by the presence of intercalary teeth on the fixed chelal finger (Mahnert 2009; Gao et al. 2018; Gao et al. 2020).

##### Distribution.

**Figure 6.**
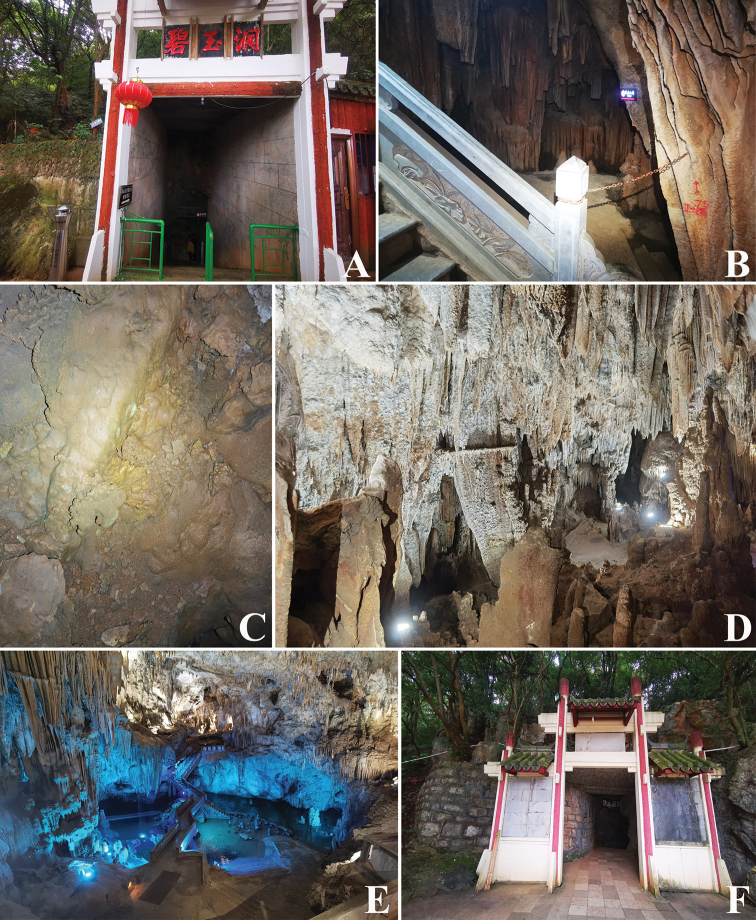
Biyu Cave, type locality of *Tyrannochthoniuspandus* sp. nov. **A** entrance **B** access to an undeveloped area **C** area where *T.pandus* specimens were collected. **D, E** beautiful cave landscapes inside the cave **F** exit.

This species is known only from the type locality, Biyu Cave (Figs 1A, 6). Biyu Cave is one of the tourist caves in the Ancient Alu Cave National Park of China, with the entrance located in the Jilong hillside. This beautiful cave is a valley type karst cave, with an internal height of 2 to 5 m and a width of 1 to 30 m. The cave has a latticed distribution. The stalactites in this cave are jasper colored, so it is called Biyu Cave (a quote from the cave’s interior slogan). The specimens of this new species were collected from under a stone and on a stone wall in an undeveloped area of the cave that is still in a natural condition. It is a small, dark, high humidity and low temperature space (temperature: 11 °C, humidity: 90%), which is suitable for the survival of the species.

#### ﻿ Lagynochthonius

Taxon classificationAnimaliaPseudoscorpionesChthoniidae

Beier, 1951

4BC57EF0-8ADE-5297-B845-C2029C55C8C4

##### Type species.

*Chthoniusjohni* Redikorzev, 1922b, by original designation.

##### Diagnosis.

See Judson (2007) and Edward and Harvey (2008).

#### ﻿ Lagynochthonius laoxueyanensis sp. nov.

Taxon classificationAnimaliaPseudoscorpionesChthoniidae

42A9FC8D-EA13-5DFD-8418-A156A6B47383

http://zoobank.org/4BD3052B-5217-4D93-B6FF-77DA99011228

**Figure 7.**
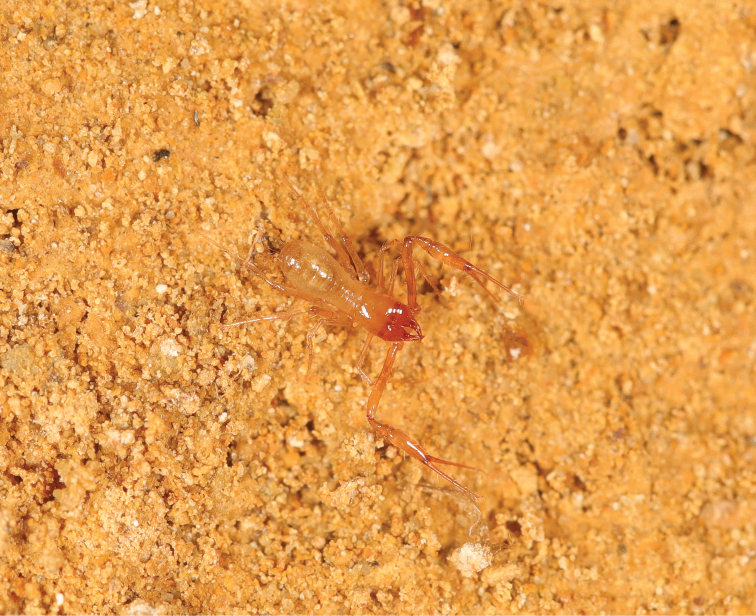
*Lagynochthoniuslaoxueyanensis* sp. nov., female habitus. Photographed when it crawled on stony natural habitat.

**Figure 8.**
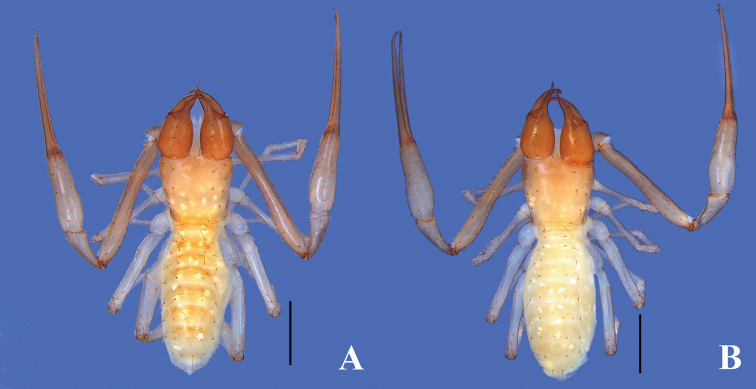
*Lagynochthoniuslaoxueyanensis* sp. nov. **A** holotype male, dorsal view **B** paratype female, dorsal view. Scale bars: 0.5 mm.

**Figure 9.**
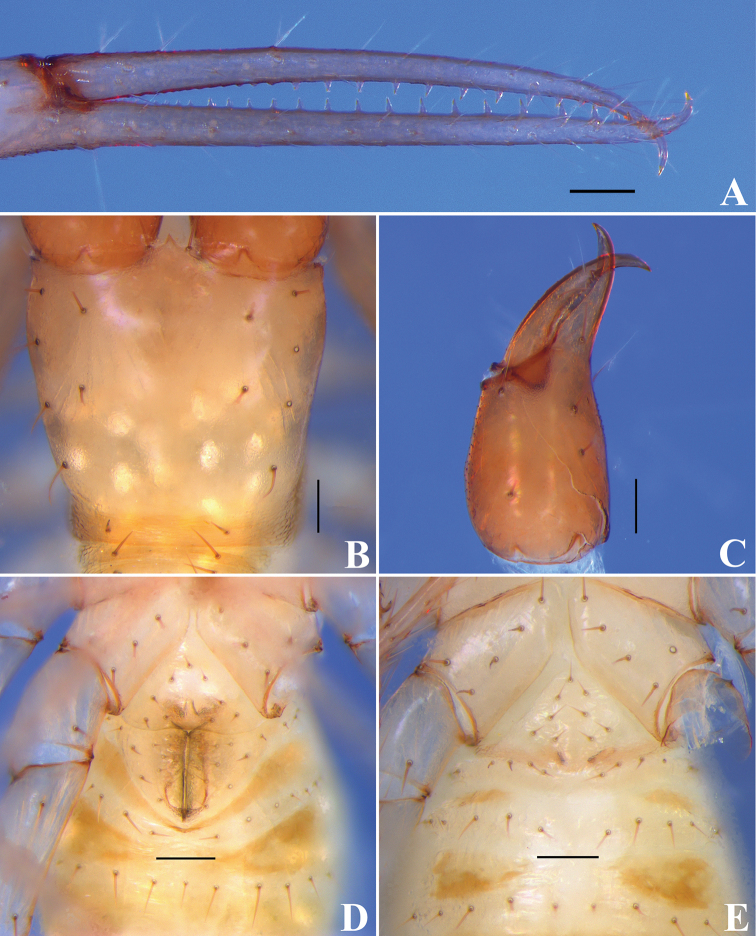
*Lagynochthoniuslaoxueyanensis* sp. nov., holotype male (**A–D**), paratype female (**E**) **A** chelal fingers (lateral view) **B** carapace (dorsal view) **C** left chelicera (dorsal view) **D** male genital area (ventral view) **E** female genital area (ventral view). Scale bars: 0.1 mm.

**Figure 10.**
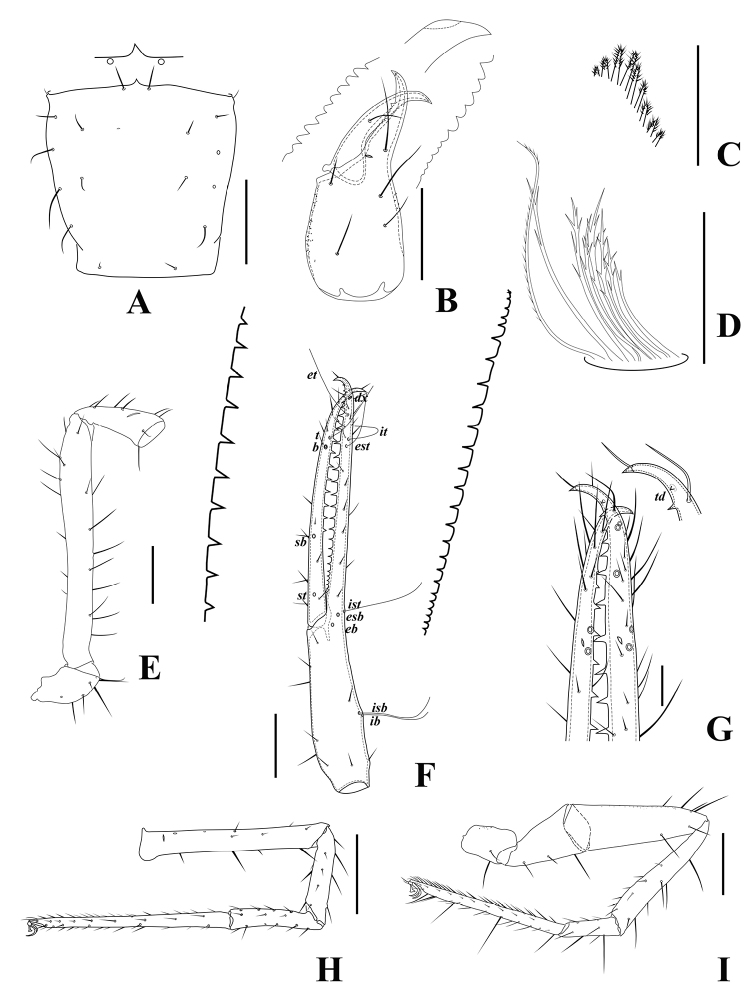
*Lagynochthoniuslaoxueyanensis* sp. nov., holotype male **A** carapace (dorsal view) with a detail of anterior margin **B** left chelicera (dorsal view) with details of teeth and tip of movable finger **C** coxal spines on coxae II (ventral view) **D** rallum **E** left pedipalp (minus chela, dorsal view) **F** left chela (lateral view) with details of teeth and with trichobothrial pattern (abbreviations explained in Material and methods) **G** finger tips of chela (lateral view) with detail of modified tooth **H** leg I without trochanter (lateral view) **I** leg IV (lateral view). Scale bars: 0.25 mm (**A, B, E, F, H, I**); 0.10 mm (**C, D, G**).

Chinese name. 老穴岩拉伪蝎 Figs 7 , 8 , 9 , 10

##### Type material.

**Figure 11.**
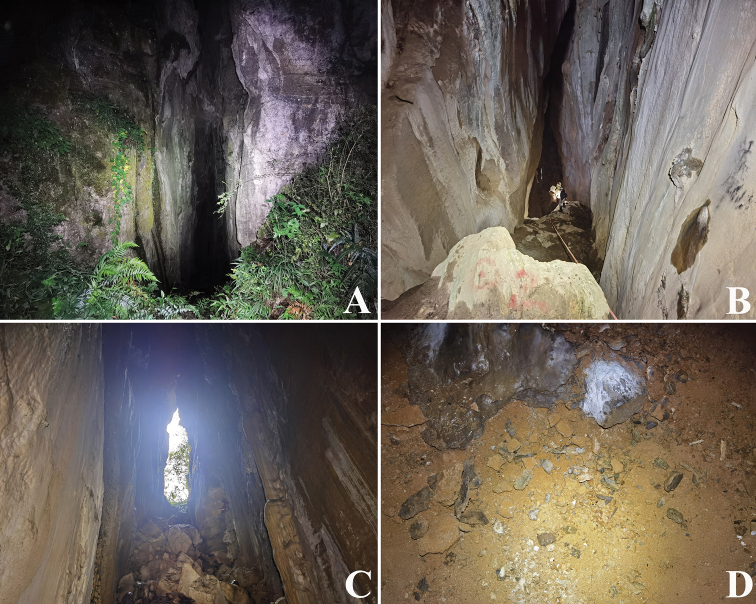
Laoxueyan Cave, type locality of *Lagynochthoniuslaoxueyanensis* sp. nov. **A** entrance **B** the only access to the deepest part of the cave **C** inside the cave entrance **D** area where *L.laoxueyanensis* specimens were collected.

(Figs 1B, 11) ***Holotype***: China • ♂; Yunnan Province, Yanshan County, Zhela Township, Liuzhao Village, Laoxueyan Cave; 23°39.03'N, 104°35.74'E; 1665 m a.s.l.; 17 Oct. 2021; Zegang Feng, Yanmeng Hou, Lu Zhang and Liu Fu leg.; dark zone; Ps.-**MHBU**-HBUARA#2021-445-01. ***Paratypes***: • 2♀; the same data as the holotype; Ps.-**MHBU**-HBUARA#2021-445-02, Ps.-**MSWU**-HBUARA#2021-445-03.

##### Diagnosis.

Moderately sized troglomorphic species with elongate appendages; carapace without eyes or eyespots; anterior margin of carapace thin, finely denticulate, epistome pointed and small, triangular; posterior margin of carapace with two setae; tergites I–II with two setae. Pedipalps slender, femur 8.54 times longer than broad; chela 7.71 times longer than broad; chela fingers gently curved in dorsal view and fixed finger with a modified accessory tooth on dorso-antiaxial face (*td*).

##### Etymology.

Latinized adjective derived from the name of the type locality, Laoxueyan Cave, Yunnan Province, China.

##### Description.

**Adult male** (Figs 8A, 9A–D, 10). ***Color***: generally pale yellow, chelicera, pedipalps and tergites slightly darker, soft parts pale (Fig. 8A). ***Cephalothorax*** (Figs 9B, 10A): carapace 1.02 times longer than broad, gently narrowed posteriorly, surface smooth, without furrows but with 1 small lyrifissure; anterior margin thin, finely denticulate; without any traces of eyes; epistome very pointed and small, triangular; with 18 setae arranged s4s: 4: 4: 2: 2, most setae heavy, long and gently curved, anterolateral setae much shorter than others (Fig. 10A). Chaetotaxy of coxae: P 3, I 3, II 4, III 5, IV 5; manducatory process with two acuminate distal setae, anterior seta less than 1/3 length of medial seta; apex of coxa I with small, rounded anteromedial process; coxae II with 9 terminally indented coxal spines on each side, set as an oblique row, longer spines present in the middle of the row, becoming shorter distally and proximally and incised for about half their length (Fig. 10C); intercoxal tubercle absent; without sub-oral seta. ***Chelicera*** (Figs 9C, 10B): large, about same length as carapace, 2.37 times longer than broad; 5 setae on hand, all setae acuminate, ventrobasal seta shorter than others; movable finger with one medial seta. Cheliceral palm with moderate hispid granulation both ventral and dorsal side. Both fingers well provided with teeth, fixed finger with 12 teeth, distal one largest; movable finger with 10 retrorse continuous small teeth; galea weakly raised, keel-like (Fig. 10B). Rallum with 8 blades, the distal one longest and recumbent basally, with fine barbules and slightly set apart from the other blades, latter tightly grouped and with long pinnae, some of which are subdivided (Fig. 10D). ***Pedipalp*** (Figs 9A, 10E–G): surface of palpal segments smooth; chelal palm gradually constricted towards fingers; setae generally long and acuminate; femur 8.54, patella 2.73, chela 7.71, hand 3.10 times longer than broad; movable finger 1.45 times longer than hand and 0.58 times longer than chela, apodeme complex of movable finger strongly sclerotized. Femur and dorsal hand without tactile setae but with 1 lyrifissure distally at patella (Fig. 10E). Fixed chelal finger and hand with 8 trichobothria, movable chelal finger with 4 trichobothria, *ib* and *isb* situated close together, submedially on dorsum of chelal hand; *eb*, *esb* and *ist* forming a straight oblique row at base of fixed chelal finger; *it* slightly distal to *est*, situated subdistally; *et* slightly near to tip of fixed finger, very close to chelal teeth; *dx* situated distal to *et*; *sb* situated closer to *st* than to *b*; *b* and *t* situated subdistally, *b* situated at same level as *est*; *t* situated distal to *est* and at same level as *it* (Fig. 10F). Microsetae (chemosensory setae) absent on hand and both chelal fingers. Sensilla absent but with 1 lyrifissure between *t* and *b*, *it* and *est*, respectively (Fig. 10G). Both chelal fingers with a row of teeth, homodentate, spaced regularly along the margin, larger teeth present in the middle of the row, becoming smaller distally and proximally: fixed finger with 24 large, erect, well-spaced teeth, without intercalary teeth; movable finger with 10 small (slightly smaller than the teeth on fixed finger), retrorse, serrated and well-spaced teeth; fixed finger also with a modified accessory tooth on dorso-antiaxial face (*td*) (Fig. 10F, G). Chelal fingers gently curved in dorsal view. ***Opisthosoma***: generally typical; pleural membrane finely granulate. Tergites and sternites undivided; setae uniseriate and acuminate. Tergal chaetotaxy I–XII: 2: 2: 4: 4: 4: 4: 4: 5: 5: 4: T2T: 0; tergites VIII and IX each with an unpaired median seta. Sternal chaetotaxy IV–XII: 13: 11: 9: 9: 9: 9: 9: 0: 2; sternites IV–X with unpaired median seta. Anterior genital operculum with 9 setae, genital opening slit-like, with 15 marginal setae on each side (Fig. 9D). ***Legs*** (Fig. 10H, I): generally typical, long and slender. Fine granulation present on anterodorsal faces of trochanter IV and patella IV. Femur of leg I 1.88 times longer than patella and with 1 lyrifissure at the base of femur; tarsus 2.23 times longer than tibia. Femoropatella of leg IV 3.76 times longer than deep; tibia 6.33 times longer than deep; with tactile setae on both tarsal segments: basitarsus 3.57 times longer than deep, with basal tactile setae (TS = 0.24), telotarsus 12.80 times longer than deep and 2.56 times longer than basitarsus (TS = 0.41). Setae of leg I (trochanter to tibia) 3: 11: 11: 14, setae of leg IV (trochanter to basitarsus) 2: 3: 6: 14: 10. Arolium slightly shorter than the claws, not divided; claws simple. ***Dimensions of male holotype*** (length/width or, in the case of the legs, length/depth in mm; ratios in parentheses). Body length 1.78. Pedipalps: trochanter 0.29/0.16 (1.81), femur 1.11/0.13 (8.54), patella 0.41/0.15 (2.73), chela 1.62/0.21 (7.71), hand length 0.65/0.21 (3.10), movable finger length 0.94. Chelicera 0.64/0.27 (2.37), movable finger length 0.34. Carapace 0.59/0.58 (1.02). Leg I: trochanter 0.17/0.11 (1.55), femur 0.60/0.07 (8.57), patella 0.32/0.07 (4.57), tibia 0.30/0.05 (6.00), tarsus 0.67/0.04 (16.75). Leg IV: trochanter 0.21/0.14 (1.50), femoropatella 0.79/0.21 (3.76), tibia 0.57/0.09 (6.33), basitarsus 0.25/0.07 (3.57), telotarsus 0.64/0.05 (12.80).

**Adult females** (Figs 7, 8B, 9E). Mostly same as males. Anterior genital operculum with 9 setae plus 10–12 setae on posterior margin. Body length 2.00–2.05. Pedipalps: trochanter 0.30–0.32/0.17–0.18 (1.76–1.78), femur 1.17/0.14–0.15 (7.80–8.36), patella 0.40–0.41/0.17 (2.35–2.41), chela 1.65–1.66/0.23–0.24 (6.88–7.22), hand length 0.66–0.70/0.23–0.24 (2.75–3.04), movable finger length 0.95–0.98. Chelicera 0.68–0.70/0.29–0.30 (2.33–2.34), movable finger length 0.36–0.37. Carapace 0.62/0.61 (1.02). Leg I: trochanter 0.18–0.20/0.13 (1.38–1.54), femur 0.61/0.07 (8.71), patella 0.32–0.33/0.07 (4.57–4.71), tibia 0.30/0.06 (5.00), tarsus 0.68–0.69/0.05 (13.60–13.80). Leg IV: trochanter 0.22–0.24/0.13–0.14 (1.69–1.71), femoropatella 0.81–0.82/0.21 (3.86–3.90), tibia 0.60–0.61/0.09–0.10 (6.10–6.67), basitarsus 0.26–0.27/0.06 (4.33–4.50), telotarsus 0.69/0.05 (13.80).

##### Remarks.

Of all *Lagynochthonius* species found in hypogean environments around the world, only three species, *L.bailongtanensis* Li, Liu & Shi, 2019 (from China), *L.typhlus* Muchmore, 1991 (from Jamaica) and *L.curvidigitatus* Mahnert, 1997 (from Spain), have no eyes, and are all highly troglomorphic species. *Lagynochthoniuslaoxueyanensis* sp. nov. is most similar to *L.typhlus* in having only 2 setae on tergites I–II, but the latter has intercalary teeth on the chelal fingers and a smaller body size (1.28 vs. 2.00–2.05 mm in females). *Lagynochthoniuslaoxueyanensis* sp. nov. can be easily separated from *L.bailongtanensis* by its smaller body size (*L.laoxueyanensis* 1.78 mm in male, 2.00–2.05 mm in females; while *L.bailongtanensis* is 2.55–2.92 mm in males, 2.72–2.95 mm in females), the number of setae on the anterior tergites (tergites I–II with 2 setae in *L.laoxueyanensis*, but 4 in *L.bailongtanensis*), the shape of epistome (pointed and small in *L.laoxueyanensis*, but obtuse and inconspicuous in *L.bailongtanensis*) and the number of setae on the pedipalpal coxa (3 setae in *L.laoxueyanensis*, but 5 in *L.bailongtanensis*). *Lagynochthoniuslaoxueyanensis* sp. nov. can be easily separated from *L.curvidigitatus* by the presence of a pair of curved chelal fingers in the latter and the number of setae on tergites I–II (*L.laoxueyanensis* with 2 and 2 setae, respectively, but *L.curvidigitatus* with 3 and 4 setae, respectively) (Muchmore 1991; Mahnert 1997; Edward and Harvey 2008; Mahnert 2011; Li et al. 2019).

##### Distribution.

This species is only known from the type locality, Laoxueyan Cave (Figs 1B, 11) which is located about 4 km southeast of Liuzhao Village (Yanshan County). The entrance of the cave is slit-shaped (18 m high and 4 m wide), and the total length of the cave is 88.5 m, and the vertical height of the cave is about 30 m. A descent access leads to the bottom of the cave. The bottom of the cave is a large space, covered with gravel, temperature around 13 °C, humidity over 90%. All of the specimens were collected from ground crevices near the end of the cave.

## Supplementary Material

XML Treatment for Tyrannochthonius

XML Treatment for Tyrannochthonius pandus

XML Treatment for Lagynochthonius

XML Treatment for Lagynochthonius laoxueyanensis
